# Application of ImageJ in Optical Coherence Tomography Angiography (OCT-A): A Literature Review

**DOI:** 10.1155/2023/9479183

**Published:** 2023-11-22

**Authors:** Masoud Rahimi, Esmaeil Asadi Khameneh, Hamid Riazi-Esfahani, Tahereh Mahmoudi, Elias Khalili Pour, Rahele Kafieh

**Affiliations:** ^1^Retina Ward, Farabi Eye Hospital, Tehran University of Medical Sciences, Tehran, Iran; ^2^Department of Medical Physics and Biomedical Engineering, School of Medicine, Shiraz University of Medical Sciences, Shiraz, Iran; ^3^Department of Engineering, Durham University, South Road, Durham DH1 3LE, UK

## Abstract

**Background:**

This study aimed to review the literature on the application of ImageJ in optical coherence tomography angiography (OCT-A) images.

**Methods:**

A general search was performed in PubMed, Google Scholar, and Scopus databases. The authors evaluated each of the selected articles in order to assess the implementation of ImageJ in OCT-A images.

**Results:**

ImageJ can aid in reducing artifacts, enhancing image quality to increase the accuracy of the process and analysis, processing and analyzing images, generating comparable parameters such as the parameters that assess perfusion of the layers (vessel density (VD), skeletonized density (SD), and vessel length density (VLD)) and the parameters that evaluate the structure of the layers (fractal dimension (FD), vessel density index (VDI), and lacunarity (LAC)), and the foveal avascular zone (FAZ) that are used widely in the retinal and choroidal studies), and establishing diagnostic criteria. It can help to save time when the dataset is huge with numerous plugins and options for image processing and analysis with reliable results. Diverse studies implemented distinct binarization and thresholding techniques, resulting in disparate outcomes and incomparable parameters. Uniformity in methodology is required to acquire comparable data from studies employing diverse processing and analysis techniques that yield varied outcomes.

**Conclusion:**

Researchers and professionals might benefit from using ImageJ because of how quickly and correctly it processes and analyzes images. It is highly adaptable and potent software, allowing users to evaluate images in a variety of ways. There exists a diverse range of methodologies for analyzing OCTA images through the utilization of ImageJ. However, it is imperative to establish a standardized strategy to ensure the reliability and consistency of the method for research purposes.

## 1. Introduction

Nowadays, image processing and analysis are widely used in different aspects of medical studies to find new biomarkers and faster methods to diagnose and treat diseases [1–4].

In the study of the retina and the choroid, optical coherence tomography angiography (OCT-A) is used widely. It is a noninvasive imaging technique that allows the visualization of blood flow within the retina and the choroid. The technique relies on the detection of motion contrast from blood cells within the vessels [5, 6]. The analysis of OCT-A images requires the use of specialized software capable of handling the large amount of data generated by the OCT-A devices [7].

One of the most used software for this task is the Fiji/ImageJ software package. Fiji is an open-source software platform that is based on ImageJ, a popular image analysis software. Fiji is designed to handle large volumes of image data, making it ideal for the analysis of OCT-A images, which can consist of thousands of individual images. The software incorporates a range of tools for image processing, analysis, and visualization, making it suitable for a wide range of applications [8, 9]. Fiji/ImageJ provides several key features that are particularly useful for analyzing OCT-A images. For example, it includes a range of filters that can be used to enhance image quality and reduce noise or remove artifacts. It also includes algorithms for detecting and segmenting blood vessels, allowing researchers to measure vessel density, vessel tortuosity, and other important parameters [8]. In addition, Fiji/ImageJ provides tools for generating 3D reconstructions of OCT-A images, which are useful for visualizing complex 3D structures within the retina and choroid. These reconstructions can help researchers to identify specific features within the tissue, such as newly formed blood vessels or areas of reduced blood flow [10]. One of the advantages of using Fiji/ImageJ for OCT-A analysis is that the software is freely available and widely used within the scientific community. This means that there is a wealth of documentation, tutorials, and plugins available, making it easier for researchers to learn the software and customize it for their specific needs [11]. The application of Fiji/ImageJ in OCT-A analysis has allowed researchers to more easily and accurately analyze the large volumes of imaging data generated by this technique [7]. The software's advanced processing and analysis tools, combined with its 3D reconstruction capabilities, have enabled researchers to identify new features within the retina and choroid, leading to a deeper understanding of ocular physiology and disease [10].

To provide clarification for experts and researchers in chorioretinal studies regarding the capabilities of ImageJ, this review outlines the use of ImageJ in processing and evaluating OCT-A images of the retina and choroid.

## 2. Methods

Search in PubMed, Google Scholar, and Scopus was performed in March 2023 in some combined search groups with the following keywords and their combination: “ImageJ,” “Fiji,” “optical coherence tomography angiography,” “Artifact,” and “Thresholding” and relevant articles from 2010 to 2023 were selected. The inclusion criteria were original articles, review articles, and case reports. Non-English articles and abstracts were excluded. After the initial search, the first screening of articles was performed by a rapid review of article topics. Selected papers underwent a second screening by reviewing their abstracts. All selected articles were reviewed completely by authors to review the application of ImageJ in OCT-A images. As this paper is a review article, taking informed consent was not relevant. The results of the search are summarized in the supplementary materials as a table.

## 3. Results

### 3.1. Removing Artifacts and Noises from OCT-A Images

**Figure 1:**
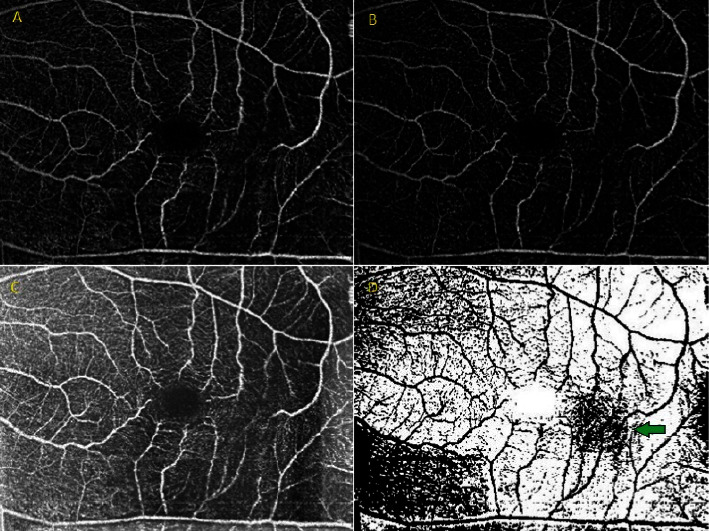
An ImageJ-processed superficial choriocapillary plexus (SCP) image of a patient presenting with a vascular disorder was utilized to enhance the visibility of the pathological vascular structure. (A) Optical coherence tomography angiography SCP image (OCT-A). (B) Implementation of a rolling ball background subtraction to enhance contrast and darkness of the background. (C) Implementation of a rolling ball background to increase the brightness of the background in preparation for thresholding the image. (D) Pathologic vessel structure, indicated by a green arrow, became visible in the thresholding image generated by the Otsu method alongside a rolling ball background subtraction.

OCT-A images are obtained by detecting the movement of blood cells in the vessels, which enables a detailed map of the vascular network to be created. However, OCT-A images often contain various artifacts and noise, which can affect the accuracy of the interpretation of the vascular images [5]. This is where ImageJ comes in, playing a crucial role in removing these unwanted artifacts [12–15] Also, it can be used to diminish noise, increase image quality, and enhance details [16–20]. ImageJ has several plugins that are designed specifically to remove artifacts and noise in OCT-A images, such as the “rolling ball background subtraction” plugin [21], the “bandpass filter” plugin [22], and the “image calculator” plugin [23]. The rolling ball background subtraction plugin works by removing the background signal in OCT-A images. The rolling ball algorithm estimates the background signal, which is then subtracted from the image, thereby enhancing contrast and improving the quality of the vascular network [24, 25] as shown in Figure 1. This plugin is particularly useful for reducing the influence of uneven illumination on OCT-A images [24].

**Figure 2:**
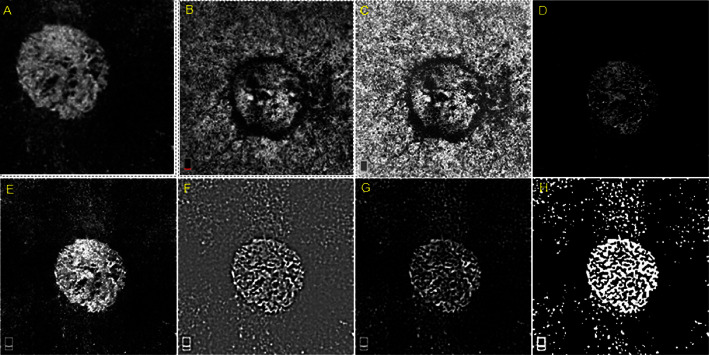
The application of ImageJ to elucidate the structure of choroidal neovascularization (CNV). Optical coherence tomography angiography (OCT-A) image of a CNV network in the outer retinal layer (A). (B) The preceding CNV in the layer of choriocapillaris (CC). (C) Use of a rolling ball background to increase the brightness of the CC image background. (D) The CNV was separated using the image calculator's subtraction mode and the combination of images (A) and (C). (E) Subsequently, employ the image calculator to combine mode images (A) and (D) in order to enhance the visibility of the CNV. (F) A bandpass filter was applied to image (D) to improve the structural view. (G) Subsequently, the image was prepared for thresholding (F) as the background became darker in the presence of a rolling ball subtraction method. Ultimately, the implementation of the ISODATA thresholding procedure elucidated the structure of the CNV.

The bandpass filter plugin is used to remove high- and low-frequency noise from OCT-A images. This plugin works by applying a filter that only keeps the frequencies within a certain range, thereby removing any noise that falls outside the desired range. The result is a clearer image with improved detail [26]. The image calculator plugin is used to combine two or more OCT-A images to create a final image with improved quality. This plugin works by subtracting one image from another or by adding one image to another [23]. In this way, it is possible to remove artifacts that are common to both images, leaving behind only the relevant information. OCT-A images are prone to motion artifacts caused by the involuntary movement of the subject or imaging system during imaging. Motion artifacts cause image distortion, loss of detail, and reduced image quality [27]. ImageJ, a free open-source image processing software, can be used to remove motion artifacts from OCT-A images. The process of removing motion artifacts from OCT-A images involves several steps, including image registration, subtraction, and filtering. Image registration involves aligning images taken at different time points to remove motion-induced misalignment. ImageJ has several plugins, including the TurboReg plugin, that can automatically align images based on a reference image [28]. Subtraction involves subtracting the registered images to remove background noise and other extraneous signals. ImageJ has several tools, including the image calculator plugin, which can perform image subtraction [29]. Filtering involves smoothing the image to reduce noise and enhance the signal. ImageJ has several filters, including the Gaussian and Median filters, that can be used to remove noise and enhance image quality [16, 30, 31]. Once the motion artifacts have been removed, the OCT-A images can be further processed and analyzed. ImageJ has several other features, including segmentation and quantification tools, which can be used to identify blood vessels and measure vessel density (VD) [32]. Overall, ImageJ is a powerful tool for removing noises and motion artifacts from OCT-A images and enhancing image quality. With the right plugins and filters, ImageJ can help researchers and clinicians obtain accurate and detailed OCT-A images. In Figure 2, the combined usage of quality improvement and thresholding is shown.

### 3.2. Thresholding Methods of OCT-A Images by ImageJ

**Figure 3:**
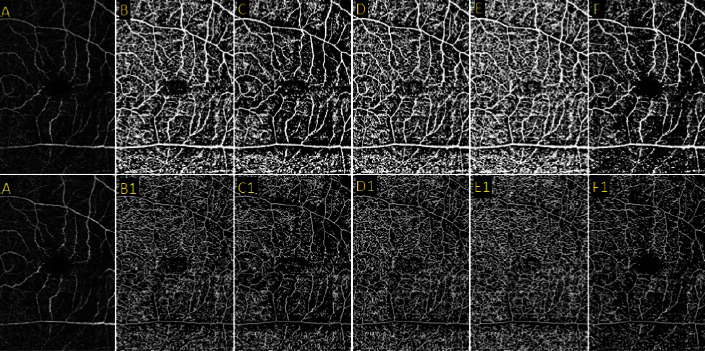
This figure illustrates the application of various thresholding techniques to an image (A). Images B, C, D, E, and F represent the binary forms of thresholding using the Phansalkar, Otsu, Niblack, mean, and default methods, respectively. Images B1, C1, D1, E1, and F1 represent the skeletonized forms derived from the corresponding binary images.

Blood vessels in the retina and choroid can be visualized with OCT-A. This technique produces volumetric data [6], and the analysis of these images can be challenging. Thresholding methods using ImageJ can help simplify this process [33]. Thresholding techniques involve setting a threshold value for the intensity of pixels in an image. This threshold value separates the image into binary regions, where pixels with intensities above the threshold are assigned one value, usually white, and pixels below the threshold are assigned another value, usually black [34]. There are three ways of thresholding images: (1) global thresholding, (2) local thresholding, and (3) complex thresholding. Global thresholding uses the same value for the entire image while local thresholding has a separate value proportional to a different zone of the image. Complex thresholding combines both global and local to build binary images [35]. The most important thing about these algorithms is to keep in mind that different algorithms cause different results in quantitative parameters significantly and make a difference in the result of the study [33, 36, 37]. These methods can be accessed through the ImageJ toolbar by selecting “image” and then “adjust threshold.” Once this window is opened, the different thresholding methods can be selected from the dropdown menu. We showed a sample of thresholding in Figure 3.

Corvi et al. compared the result of different thresholding methods on the amount of VD. They compared five methods and finally, as a result, mentioned that the VD amount of each thresholding method (to make binary images) is different from one another significantly. In their study, the amounts of Otsu and ISODATA methods were close to each other [38]. Also, these algorithms are sensitive to images' different contrasts which can be affected by the process of capturing or exporting images [33]. In order to have standard quantitative data, it seems better to make unification in choosing the best algorithms in studies because it is impossible to compare the results when the methods and data of studies are different. Some studies suggest local thresholding in order to make binary images [34, 39, 40]. Laiginhas et al. in the study of choriocapillaris evaluated different thresholding methods to assess their repeatability: (1) the local method (Niblack, mean, and Phansalkar) and (2) the global method (default, mean, and Otsu). To compare the methods, they used fellow deficit items (density, mean size, total area, and number) as quantitative parameters. As a result, they mentioned that the local methods are more repeatable and better than the global ones [39]. William et al. in another study used VD because of its huge usage in chorioretinal studies as a parameter to compare different thresholding methods. They first removed the artifacts and noises from the images. Then, three of the authors manually binarized the images blindly. Finally, they obtained VD from the images. After that, they used three ways to binarize images (one step, two steps, and three steps). The higher the steps, the more the filters used to reduce the noise. Finally, after analysis of the results, they mentioned that the bandpass filter plus Phansalkar (local) threshold (two steps thresholding) is the best way to binarize images [34]. After the threshold is applied, the binary image can be further processed to remove noise or fill in any gaps in the segmented vessels. This can be accomplished using morphological operations such as erosion, dilation, opening, and closing, which are also available in ImageJ.

As a result, thresholding methods using ImageJ could be useful in segmenting and analyzing OCT-A images. These methods can be combined with morphological operations to further refine the binary images and obtain accurate measurements of vessel density and diameter.

### 3.3. Structural Parameters (Fractal Dimension, Vessel Diameter Index, and Lacunarity)

The fractal dimension (FD) is another crucial parameter in chorioretinal research. It is a biomarker used to assess the chorioretinal microvascular structure [41–47] and may be extracted from binarized and skeletonized OCT-A images using ImageJ [48]. With skeletonized images, the algorithm is more sensitive to vascular alterations [49]. In addition, it is extremely useful for measuring retinal vascular activity [48, 49] and the complexity of branching vascular networks [50]. The greater the FD, the denser the vascular structure [48, 49]. Patients with certain retinal diseases, such as age-related macular degeneration (AMD) and diabetic retinopathy (DR), can benefit from the application of FD in diagnosis and monitoring [49, 51]. The typical value for the FD range of the chorioretinal vasculature in human eyes is 1-2. This number characterizes the amount of space filled by chorioretinal vascular branches or the complexity of vascular branching [52]. Different thresholding methods result in different amounts of FD as a sample shown in Table 1. There are four methods to compute FD (correlation dimension, box-counting dimension, generalized dimension, and information dimension). Before calculations can be performed, images must be binarized. Counting-box dimension (Dbox) is the most widely used method for calculating fractal dimension (FD) in previous studies [53]. ImageJ employs a grid and counts the grid's boxes in each portion of the vascular pattern [54]. Dimensional generalization is beneficial for multifractal structures. Instead of counting boxes, software in the information dimension assigns a value to each one and then adds up all the values. Although FD is often used for the chorioretinal vasculature as a whole, it is also used independently for veins and arteries in the reviewed literature. FD relies on a number of variables (region of interest, imaging method, image processing, FD analysis tool, and kind of vessel) as they pertain to the creation of binary images [53]. In one investigation on diabetic individuals with DR, when FD was compared globally with healthy eyes, there was no difference between the two groups; however, when FD was evaluated locally, it was reduced in DR [55]. It appears that FD analysis needs methodological standardization in order to eliminate bias in research and produce comparable findings. Qian Xu et al. analyzed the effects of hypertension on the arteries of the retina in OCT-A images. They compared VD and FD between patients and controls. Lastly, they noted that VD and FD were decreased in patients as a result of hypertension [56]. Fayed et al. worked on DR by creating binarized images from superficial choriocapillary plexus (SCP) OCT-A images. They noted that FD is lower in DR than in the normal population. Moreover, FD can be used to monitor people without clinical manifestations of DR [57].

Another biomarker that can be utilized in chorioretinal research is the vessel diameter index (VDI), which can evaluate vascular morphology and alterations in vascular structure [44, 58–66]. It may be derived by Fiji from both binarized and skeletonized OCT-A images [62]. It displays the average vessel diameter or caliber [64] and the relationship between vessel diameter and length [67]. In other words, it is the relationship between VD and skeletonized density (SD) [46]. Kim et al. investigated the changes in VD, VDI, SD, and FD in uveitis patients and compared them with healthy eyes. VD, SD, and FD were considerably decreased in both the superficial and deep layers compared to healthy eyes, whereas VDI was unchanged [66]. Mastropasqua et al. evaluated several biomarker variations following vitreoretinal surgery in order to determine vascular changes in the idiopathic epiretinal membrane (iERM). They estimated vessel length density (VLD) based on skeletonized OCT-A images acquired from binary images. In addition, VDI was measured to determine the mean vessel caliber. As a consequence, they noted that VLD and VDI improved dramatically after six months of follow-up, which is consistent with patients' recovery [61].

Lacunarity (LAC) is an additional biomarker that depicts the spatial dispersion of blood vessels [63]. It is another morphologic biomarker (like FD and VDI) obtained from skeletonized images in chorioretinal studies [41–44, 47, 59, 68]. A significant amount of LAC reflects heterogeneous vasculature, whereas a low amount of LAC reflects homogeneous vasculature [69]. In other words, it reveals the spaces between vessels. The larger the LAC, the wider the gaps [70]. Depending on the vessel shape, LAC is smaller than FD and can be used to eliminate discrepancies across images with equal FD. Using both LAC and FD to evaluate the geometry of the chorioretinal vascular system is more specific than using FD alone [71]. FracLac is a plugin that is used to calculate FD and LAC and other structural parameters in ImageJ [72]. In a 2019 study, the variations in vascular geometry between healthy and diseased retinas were evaluated. In healthy eyes, LAC and VD were lower in the macular area than in the optic disc zone, while FD was the same. LAC was raised in DR, although FD and VD remained unchanged [55]. In several additional research studies on DR, FD was reduced and LAC rose as a result [71, 73]. FD has increased in the other trials as well [74]. These differences in outcome may be attributable to the methods and processes used. As stated earlier, it is essential to employ a comparable method in order to obtain comparable outcomes. Ouederni et al. conducted research on the patients with retinal vein occlusion (RVO). They processed and analyzed OCT-A images using ImageJ. Initially, VD was extracted from binarized images, VDI was extracted from both skeletonized and binarized images, and SD, LAC, and FD were extracted from skeletonized images. Then, they compared them between groups of patients and healthy individuals. Therefore, they reported that FD, SD, and VD were lowered in patients, while VDI and LAC were enhanced. Lastly, they proposed FD and SD as novel deep choriocapillary plexus (DCP) parameters for predicting visual function in RVO [59]. Popovic et al. evaluated the retinal microvascular morphologic alterations in DR. First, they subcategorized images based on the severity of DR and then employed FD and LAC to measure the changes. The severity of DR was shown to be linked with these biomarkers. In these instances, by increasing the severity of DR, FD was raised while LAC was lowered. Finally, they proposed this strategy as a noninvasive technique for detecting severe DR [47].

### 3.4. Perfusion Parameters (Vessel Density, Skeletonized Density, and Vessel Length Density)

One critical feature of OCT-A images is measuring the vascular density, which is the number of pixels representing blood vessels divided by the total pixels in the analysis area [34, 75]. Vascular density is a vital biomarker in ophthalmology, as it provides information on retinal perfusion and is associated with various ocular diseases such as diabetic retinopathy, glaucoma, and age-related macular degeneration [49, 76, 77]. ImageJ can be used for various image analyses, including measuring the vessel density in OCT-A images. The following are some methods for measuring vascular density in OCT-A using ImageJ.

#### 3.4.1. Manual Threshold Segmentation

Manual threshold segmentation is the simplest and most widely used method for analyzing OCT-A images. This method involves manually selecting a threshold value to separate the vessel regions from the background regions. The user can modify the threshold value to optimize the sensitivity and specificity of the analysis. However, this method is tedious and time-consuming, and the results can vary depending on the user's skills and judgment [78].

#### 3.4.2. Semiautomatic Threshold Segmentation

**Figure 4:**
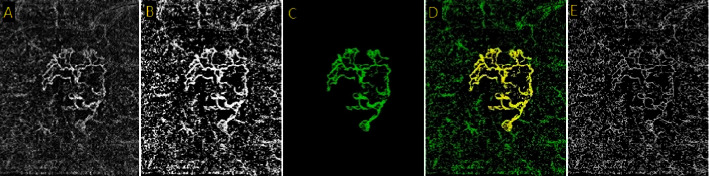
To determine the vessels and delineate their boundaries in preparation for analysis, ImageJ is utilized. In the choriocapillaris (CC) layer, an OCT-A image identified a choroidal neovascularization (CNV) complex (A). It was binarized utilizing the contrast thresholding technique (B). Subsequently, the outer portion of the CNV was eliminated by selecting its border (C). By integrating images (B) and (C), the CNV region was rendered (D) amenable to fractal dimension and vessel density analysis. The image must be in skeletonized form in order to derive additional parameters such as vessel length density. In conclusion, the skeletonized form was constructed using the binary image (E).

Semiautomatic threshold segmentation is an improved version of manual threshold segmentation that involves using an algorithm to segment the images. The algorithm automatically selects an optimal threshold value based on the image histogram analysis. This method is more objective, time-efficient, and accurate than manual threshold segmentation. However, the algorithm's performance depends on the underlying image characteristics and the abovementioned thresholding methods [78]. Two forms of density are employed in the analysis of OCT-A images: vascular density (VD) and SD. They are the most common biomarkers in OCT-A image analysis [79]. These indicators can both be used to evaluate vascular perfusion. Many research studies calculated the VD of OCT-A images using ImageJ [14, 18, 41, 45, 49, 56, 58–60, 62, 63, 65, 66, 77, 80–83]. VD and vascular length (obtained from binarized and skeletonized images) are the most prevalent biomarkers in OCT-A image analysis [79].  Figure 4 shows a sample of image processing and analysis with ImageJ to calculate the VD in a patient with choroidal neovascularization.

VD is the proportion of pixels occupied by the blood vessel (or blood flow) relative to the whole pixels of the image [34, 77]. VD corresponds to the FD and can show microvascular perfusion changes and blood flow area and detect microvascular diseases [75]. Similar to FD, the quantity of VD obtained from binarized images is dependent on the thresholding method [37, 84]. In addition, many OCT-A systems do not provide the same VD value for a single subject [82, 85–88]. Yang et al. reported that the quantity of VLD and VD in SCP and DCP varies in OCT-A devices based on imaging technique and scan pattern [82]. Lee et al. investigated patients with RVO and the repeatability of VD and foveal avascular zone (FAZ) measurement. They noted that VD in the SCP layer is reproducible but is not significantly repeatable in the DCP layer. In addition, they highlighted that the image quality affects the repeatability of the VD measurement. In contrast, manual measurement of FAZ was highly reproducible in both SCP and DCP layers [89]. Rabiolo et al. investigated several OCT-A system angiocubes. They evaluated the repeatability of VD and FAZ area throughout all retinal layers. They concluded that comparing perfusion parameters in images of the same angiocube size is preferable since VD varies dramatically across image sizes. Nevertheless, FAZ was more consistent across sizes, and lower scan sizes were preferable [90]. A research found that high-resolution and high-speed modes of OCT-A systems resulted in varied VD and VLD measurements across all retinal layers [88]. Levine et al. evaluated the repeatability and reproducibility of VD and SD in four OCT-A devices. They noticed that the repeatability of VD is more dependable than that of SD between various instruments, but it was not identical in all cases. In both devices, the repeatability of VD in the SCP and DCP layers was quite similar but not identical. SD was absolutely unreproducible across all devices and retinal layers [87]. SD denotes the proportion of the total vessel length in one-pixel width inside the evaluation region. Due to its width and decreased weight of large vessels, it is more sensitive to microvascular alterations and is unaffected by poor image quality compared to VD [91]. According to one study, the quantity of SD such as VD would vary depending on the processing method [84]. In this study, a sample of different amounts of them is shown in Table 1. Other studies obtained this information using skeletonized OCT-A images [46, 56, 58, 59, 62, 66, 92]. Hsiao et al. investigated the relationship between the number of OCT-A biomarkers and visual acuity in diabetic macular edema (DMO). They extracted FD, VD, and SD from OCT-A images using ImageJ. The researchers then compared and examined the amounts of each biomarker. They concluded that FD and SD in DCP alone are considerably lower in patients with poor best-corrected visual acuity (BCVA) [46]. Phasukkijwatana et al. investigated birdshot chorioretinopathy (BCR). For quantitative analysis of the OCT-A images, they utilized ImageJ. Images were binarized and skeletonized for analysis purposes. Later, they assessed VD using ImageJ, which is more sensitive to alterations in tiny vessels and demonstrated that the blood flow of SCP and DCP is decreased in this condition. DCP changes exceeded SCP changes. As they used Fiji to minimize the artifacts, DCP modifications were more significant [14]. In the second research, Cheng et al. evaluated the efficacy of RVO on VD. They determined all three levels of retinal VD using ImageJ. In addition, they employed this technique to measure the treatment's efficacy by assessing the changes in vessel density after following patients. As a result, they mentioned that the treatment of RVO with antivessel endothelial growth factor can help to decrease VD of the macula [81].

VLD is the total vascular length of pixels per unit area in a skeletonized image evaluation zone [93]. Researchers have employed VLD shifts as a diagnostic and differential measure in several studies [17, 44, 45, 61, 65, 82, 94]. In the transition from a binarized image to a skeletonized image, the vessel caliber pixels are reduced to a single pixel, hence preventing the influence of vessel size on VLD [94]. Li et al. analyzed the influence of axial length (AL) and scanned area on the quantity of VLD. They determined that the quantity of VLD will vary between AL [93]. Arias et al. attempted to identify biomarkers that aid in the early detection of microvascular alterations in prediabetic individuals. They processed and analyzed the OCT-A images using ImageJ. VLD was extracted from skeletonized images. They concluded that VLD is considerably reduced in diabetic and prediabetic patients compared to the control group in both SCP and DCP [94].

### 3.5. Foveal Avascular Zone

The FAZ is a circular region devoid of vessels in the foveal area. It plays a crucial role in visual processes such as central vision and the blood supply to the macula. Alterations to the shape and size of the FAZ can be used as indicators in patient diagnosis and follow-up [95]. In some instances, such as prematurity retinopathy [96], it can be smaller; however, in ischemic diseases such as DR and RVO [97, 98], it can expand. FAZ varies in size and shape depending on the individual [99]. FAZ borders can be selected manually (plot all vascular endpoints) and automatically. While the manual segmentation of FAZ has demonstrated its reliability in producing consistent outcomes [89, 100], it is not without limitations. One such disadvantage is its reliance on the operator, which introduces the potential for differences in the findings. Additionally, the manual approach becomes impractical when dealing with extensive datasets and numerous images. Thus, employing automated approaches such as ImageJ can decrease time and mistakes [7]. The FAZ region may also be derived via OCT-A devices; however, several studies have noted its limitations and flaws [101–103]. Many investigations have found that the quantity of FAZ in various angiocubes is consistent [90, 104]. Shiihara et al. examined the FAZ determination findings of three OCT-A devices, and they reported that the quantity of each device differs from that of the others. Thus, the disparity between the results of each device renders them difficult to compare or incomparable [105]. Lin et al. evaluated two ImageJ macros (level sets macro (LSM) and Kanno–Saitama macro (KSM)) using a Cirrus OCT-A device to discover which is the most effective for determining FAZ metrics such as area, circularity, and perimeter. In comparison to the two macros, Cirrus OCT-A has some bias in estimating the FAZ dimension. Lastly, they provided the intraclass correlation coefficient (ICC) and coefficient of variation (CoV) for each algorithm and manual technique. In their investigation, LSM (ICC: 0.908; CoV: 9.664%) and the manual approach (ICC: 0.963; CoV: 6.109%) were superior to the other methods and were comparable, whereas Cirrus OCT-A performed poorly (ICC, 0.603; CoV, 27.798%), and KSM performed significantly better (ICC, 0.789; CoV, 15.788%) [106]. Another investigation found that Cirrus OCT-A was 22.9% inaccurate in identifying the FAZ boundary [101]. In another investigation, the repeatability of several parameters across many OCT-A devices was evaluated. As a result, neither VD nor SD could be reproduced in SCP or DCP layers. FD was only repeatable in the SCP layer, but it was distinct in the DCP layer. FAZ was considerably repeatable in all layers, indicating a similar parameter amongst the devices even when the other parameters were different [85].

## 4. Conclusion

In this review, we presented the application of ImageJ in the analysis of OCT-A images. ImageJ could help to improve OCT-A image quality by removing different kinds of imaging artifacts. Thresholding methods can help in segmenting and analyzing OCT-A images and also can be combined with other morphological parameters to refine the binary images and make better and more accurate biomarkers such as VD. ImageJ is also helpful for better visualization and delineation of retinal vascular pathologies. Our review showed that quantitative parameters such as VD, VDI, SD, VLD, FD, and LAC could help to differentiate between different retinal vascular diseases. To date, there is not a single standardized protocol for analyzing OCT-A images with ImageJ, so further studies are needed. In the future to reach a common way of study and have comparable results in analyzing OCT-A images with ImageJ, it is needed that studies try to find the best way and the most reliable way of analyzing images with ImageJ.

## Figures and Tables

**Table 1:** The amount of fractal dimension (FD) (obtained from Figure 3), vessel density (VD), and vessel length density (VLD) (obtained from Figure 4) in a single image with different binarized and skeletonized methods.

Method	Figure 3		Figure 4	
	Skeletonized FD	Binarized FD	VD	VLD
Phansalkar	1.9474	1.9236	58.093	19.603
Otsu	1.9474	1.9311	53.989	17.471
Niblack	1.9474	1.9240	46.521	17.127
Default	1.9475	1.9327	53.989	17.471
Mean	1.9473	1.9151	46.651	22.714

## Data Availability

The data used to support the findings of this study are available from the corresponding author upon request.

## Supplementary Materials

Supplementary Materials In March 2023, PubMed, Google Scholar, and Scopus were searched using the following keywords and combinations: “ImageJ,” “Fiji,” “optical coherence tomography angiography,” “Artifact,” and “Thresholding” to find relevant articles from 2010 to 2023. Original, review, and case reports were accepted. Non-English articles and abstracts were eliminated. After the initial search, article subjects were quickly screened. Authors investigated all chosen studies to evaluate ImageJ in OCT-A images. The table shows search results.
