# Supplementary material for: Application of ImageJ in Optical Coherence Tomography Angiography (OCT-A): A Literature Review
Source: J Ophthalmol. 2023 Nov 22;2023:9479183. doi: 10.1155/2023/9479183 (PMC10686712; doi:10.1155/2023/9479183)
Supplement: Supplementary Materials — In March 2023, PubMed, Google Scholar, and Scopus were searched using the following keywords and combinations: “ImageJ,” “Fiji,” “optical coherence tomography angiography,” “Artifact,” and “Thresholding” to find relevant articles from 2010 to 2023. Original, review, and case reports were accepted. Non-English articles and abstracts were eliminated. After the initial search, article subjects were quickly screened. Authors investigated all chosen studies to evaluate ImageJ in OCT-A images. The table shows search results. [file 9479183.f1.docx]

**Supplements:**

**Supplemental Table:**

The result of search in databases

| **Database** | **Total number or records** | **Number of excluded articles** | **Number of included articles** |
| --- | --- | --- | --- |
| PubMed | 113 | 31 | 82 |
| Scopus | 108 | 16 | 92 |
| Scholar | 169 | 37 | 132 |

**Supplemental Figure 1:**

1. Image> type> 8-bit
2. Process> Subtract Background> Radius 10 pixels and dark background
3. Process> Subtract Background> Radius 40 pixels and light background
4. Image> Adjust> threshold> Otsu> light background

**Supplemental Figure 2:**

1. Image> type> 8-bit (for both Outer retina (OR) and choriocapilary (CC) image)
2. CC image: Process> Subtract Background> Radius 50 pixels and light background
3. Process> Image Calculator> Subtract OR and CC
4. Process> Image Calculator> Add OR and the result of pervious step
5. Process> FFT> Bandpass Filter> Filter large 4 pixels, and Filter small 3 pixel
6. Process> Subtract Background> Radius 5 pixels and dark background
7. Image> Adjust> threshold> IsoData> dark background

**Supplemental Figure 4:**

1. Image> type> 8-bit
2. Image> Adjust> Auto Local Threshold> Contrast
3. Polygon Selections
4. Edit> Selection> Fit Spline
5. Edit> Selection> Area to Line
6. Edit> Clear Outside
7. Image> Color> Merge Channels> C1 and C2
